# Selected-Lesion Stereotactic Radiosurgery (SL-SRS) as a Novel Strategy in the Treatment of Patients With Multiple Brain Metastases

**DOI:** 10.7759/cureus.45457

**Published:** 2023-09-18

**Authors:** Brianna C Theriault, Charu Singh, James Yu, Jonathan Knisely, Matthew Shepard, Rodney E Wegner, Ronald E Warnick, Selcuk Peker, Yavuz Samanci, Daniel M Trifiletti, Cheng-chia Lee, Huai-che Yang, Kenneth Bernstein, Douglas Kondziolka, Manjul Tripathi, David Mathieu, Georgios Mantziaris, Stylianos Pikis, Jason Sheehan, Veronica L Chiang

**Affiliations:** 1 Neurosurgery, Yale School of Medicine, New Haven, USA; 2 Radiation Oncology, Yale School of Medicine, New Haven, USA; 3 Radiation Oncology, St. Francis Hospital, Trinity Health of New England, Hartford, USA; 4 Radiation Oncology, Weill Cornell Medicine, New York, USA; 5 Neurosurgery, Allegheny Health Network, Pittsburgh, USA; 6 Radiation Oncology, Allegheny Health Network, Pittsburgh, USA; 7 Neurosurgery, Jewish Hospital Mayfield Clinic, Cincinnati, USA; 8 Neurosurgery, Koç University School of Medicine, Istanbul, TUR; 9 Neurosurgery, Koç University Hospital, Istanbul, TUR; 10 Radiation Oncology, Mayo Clinic, Jacksonville, USA; 11 Neurosurgery, Taipei Veterans General Hospital, New Taipei, TWN; 12 Radiation Oncology, NYU Langone Medical Center, New York, USA; 13 Neurosurgery, NYU Langone Medical Center, New York, USA; 14 Neurosurgery, Postgraduate Institute of Medical Education and Research, Chandigarh, IND; 15 Neurosurgery, Université de Sherbrooke, Sherbrooke, CAN; 16 Neurosurgery, University of Virginia School of Medicine, Charlottesville, USA

**Keywords:** neuro oncology, central nervous system metastasis, brain met, gamma knife (gk) radiosurgery, radiation oncology neurosurgery, neurosurgery oncology, stereotactic radiosurgery srs

## Abstract

Introduction: With the diminishing use of whole-brain radiotherapy (WBRT), there is increasing debate regarding the maximum number of brain metastases that should be treated with stereotactic radiosurgery (SRS). In patients with >10-15 lesions, some groups are proposing a new approach - selected-lesion SRS (SL-SRS) - where only a subset of intracranial lesions are chosen for irradiation. This study is an initial look into this practice.

Methods: This is a cross-sectional exploratory survey study. A survey of 19 questions was created by the International Radiosurgery Research Foundation (IRRF) using open-ended and multiple-choice style questions on SL-SRS practices and indications with the goal of qualitatively understanding how SL-SRS is being implemented worldwide. The survey was distributed to physicians in the United States (US) and internationally who are members of the IRRF and who perform SRS frequently. Ten out of 50 IRRF institutions provided responses reflecting the practices of 16 physicians.

Results: SL-SRS is being performed at 8/10 institutions. The most common reasons for using SL-SRS included patients with prior WBRT, patients with progressing systemic disease with central nervous system (CNS)-penetrating or immunotherapies available, specific requests from medical oncology, and cooperative studies using this approach. Lesion size was cited as the most important factor when choosing to irradiate any single lesion. The majority of respondents reported 30 mm and 40 mm as size cutoffs (by largest dimension) for treatment of a lesion in eloquent and non-eloquent locations, respectively. Eloquence of lesion location and attributable symptoms were also considered important. Progression of untreated lesions was the most common reason reported for bringing patients back for additional treatment.

Conclusion: The responses to this survey show that SL-SRS is being used, allowing for small/asymptomatic brain metastases to be left safely unirradiated. It is currently used in patients who have >10-15 lesions with prior WBRT, those with progression of extracranial disease but with acceptable systemic treatment options, and those with poor functional status. The incorporation of this new approach into clinical trials should be considered for the safe study of the efficacy of new CNS-penetrating systemic therapies.

## Introduction

Intracranial stereotactic radiosurgery (SRS) has become the mainstay initial and salvage treatment for most patients with brain metastases, both singular and multiple [1-2]. SRS offers both effective local control and fewer side effects compared with whole-brain radiotherapy (WBRT) [3]. Most evidence supports the use of SRS for up to 10-15 brain metastases [4-7]. However, some argue that all brain metastases (regardless of number) should be treated with SRS [8-10]. Ultimately, the cutoff number for metastatic lesions treatable using SRS varies across institutions and is often influenced by the patient's functional status, expected duration of patient survival, local technological capability, and patient and physician level of concern about WBRT toxicity.

Given the trend away from using WBRT [2], there has been increasing discussion of an alternate treatment strategy for patients with >10-15 brain lesions by targeting only a select subset of the imaging-defined tumors with SRS - so-called selected-lesion SRS (SL-SRS). The rationale behind this strategy is that multiple brain metastases usually reflect uncontrolled systemic disease with a likely poor prognosis. SRS would, therefore, only be used to treat symptomatic lesions or lesions at “high risk” for becoming symptomatic while the efficacy of a new systemic therapy is tested - leaving small and “low risk” lesions to be observed and irradiated later if needed if the patient had a good systemic outcome.

Traditionally, this approach was considered in patients who had prior WBRT and who presented with >10-15 metastases. In order to avoid repeat WBRT and its risk of neurotoxicity [3,11-14], SRS was considered for the treatment of only larger, rapidly progressive, or symptomatic lesions thereby constituting SL-SRS.

More recently, SL-SRS has been proposed in patients without prior WBRT treatment in the context of newer and more available systemic therapy options with some degree of central nervous system (CNS) penetration. With this treatment philosophy, asymptomatic brain metastasis can, therefore, be left unirradiated and instead monitored radiographically as a patient continues systemic therapy. As it currently stands, this approach is in direct contradiction to the standard approach to treatment of CNS metastatic disease. As such, there is no prospective literature to guide SL-SRS practices, including indications for SL-SRS and which lesions should be chosen for treatment. Therefore, we surveyed clinicians skilled in SRS at high-volume centers on their experience and approach to SL-SRS, including factors they used to decide how many and which lesions to treat.

## Materials and methods

This was a cross-sectional exploratory survey study developed by members of the International Radiosurgery Research Foundation (IRRF) with the goal of understanding current opinions on SL-SRS. The IRRF is a non-profit scientific and educational entity consisting of neurosurgeons, radiation oncologists, and medical physicists at academic and clinical centers that perform SRS, track outcomes, and develop research proposals and clinical trials surrounding SRS. Membership to the IRRF is reviewed by a Board of Directors. The 19-question survey was generated by IRRF members and distributed via e-mail to all IRRF-participating centers (Table 1). Participation in IRRF-generated projects is optional to all IRRF members. IRRF members respond to IRRF-generated projects if they have applicable data.

**Table 1:** IRRF-Generated Survey An IRRF-generated survey was distributed to 50 different SRS institutions worldwide. Possible answer choices were provided when applicable (including Yes/No questions and multiple choice). Questions were otherwise open-ended. Abbreviations: Q – question, SRS – stereotactic radiosurgery, GK – gamma knife, LINAC – linear accelerator, CNS – central nervous system, WBRT – whole brain radiotherapy, ECOG - Eastern Cooperative Oncology Group, KPS – Karnofsky Performance Score.

	Question	Answer choices		Question	Answer choices
Q1	Is your center located in the USA? If not, in which country are you located?		Q11	Are there any established clinical indications for selected lesion SRS at your institution?	Y/N
Q2	Total number of cases treated with radiosurgery at your institute last year (2021).		Q12	Are there research protocols in your institution that would take priority over a selected lesion approach for patients with multiple brain metastases?	Y/N
Q3	Number of brain metastases patients treated with radiosurgery per year		Q13	Are there clinical trials at your institution that specifically request selected lesion SRS in order to study the effect of a drug in CNS?	Y/N
Q4	Does your institution have a formal or informal upper limit on the number of brain metastases treatable with SRS (in 1-5 fractions)?	Y/N	Q14	In a patient with biopsy-proven carcinoma and >15 previously radiosurgically treated brain metastases, please mark all the factors that would encourage you to consider the selected lesion SRS.	a) Age <50 b) Patient good functional status (ECOG 0/1 or KPS>70) c) Radioresistant pathology d) Refusing WBRT e) Prior WBRT f) One prior SRS treatment within 3 months g) One prior SRS treatment >1 year previously h) Multiple prior SRS treatments i) Lesions located in critical locations e.g. brainstem j) Symptomatic brain metastases amenable to SRS k) Stable systemic disease with >20 brain metastases l) Progressing systemic disease & no good standard systemic therapy options available m) Progressing systemic disease & CNS penetrating drug option available n) Progressing systemic disease & immunotherapy option available o) Progressing systemic disease & clinical trial options available p) Other
Q5	If yes to Q4, then what is the upper limit number?		Q15	Rank in order of importance the factors that your center would use to choose any single lesion for SRS if offering selected lesion SRS	a) Lesion location b) Lesion size c) Distance from any prior SRS d) Focal Signs/ Symptoms e) Perilesional edema
Q6	What percentage of your SRS-treated brain metastases patients had this # of lesions treated at the time of SRS? Each treatment should be considered a new instance for those patients who had two or more SRS sessions in the calendar year 2021.	a) 1-4 b) 5-10 c) 11-15 d) 16-20 e) 21+	Q16	In addition to the medical indications, which of the following practice indications might sway you to agree to selected lesions SRS:	a) Specific request by medical oncology b) Current center logistical capabilities of SRS treatment c) Insurance approval for a limited number of lesions d) Patient reliability e) Cooperative studies in this topic f) Other
Q7	Which machine(s) does your institute use for SRS?	GK and/or LINAC SRS	Q17	What is your cutoff size (largest dimension, or volume) for SRS for lesions in eloquent areas such as the brainstem or motor cortex?	a) 5mm/0.065cc b) 10mm/0.52cc c) 20mm/4.19cc d) 25mm/8.18cc e) 30mm/14.14cc f) No cutoff
Q8	Percentage of cases treated with frame or mask-based immobilization or combination.		Q18	What is your cutoff size for SRS for lesions in non-eloquent areas (largest dimension, or if volumetric cut off, diameter of equivalent circle)?	a) 10mm/0.52cc b) 20mm/4.19cc c) 25mm/8.18cc d) 30mm/14.14cc e) 40mm/33.51cc f) No cutoff
Q9	Percentage of cases treated with single fraction SRS, hypofractionated SRS, combined single fraction/hypofractionation.		Q19	If selected lesion SRS was performed, what factors would drive you to bring patients back for treatment of the rest of the lesions (Y/N)?	a) Progression of other lesions b) Improvement in functional status c) Stabilization of systemic disease d) Stabilization of CNS disease e) Other
Q10	Does your institution use selective lesion SRS (treatment of some but not all newly identified, previously untreated metastatic lesions)?	Y/N			

Types of questions included in the survey included open-ended (Question (Q)2, Q3, Q5, Q6, Q8, Q9), yes/no (Q1, Q4, Q10, Q11, Q12, Q13), multiple choice with single response only (Q17, Q18), multiple choice with unlimited responses (Q7, Q14, Q16, Q19), and one ranking question (Q15). For Question 14, respondents were presented with a hypothetical case of a patient with a biopsy-proven carcinoma with >15 previously radiosurgically-treated brain metastases and asked to designate factors that would encourage them to consider SL-SRS with no limit on the number of factors they could choose. For Question 15, respondents were asked to rank five factors influencing their decision to treat a specific lesion with radiosurgery. Rankings of 1-2 were classified as “most important,” 3-4 as “moderately important,” and 5 as “not important.” Descriptive statistics (i.e., ranges and means) were calculated for questions that solicited numerical responses where appropriate. However, no inferential statistics were performed.

## Results

Ten out of 50 institutions responded to the survey, reflecting a total of 16 clinicians across the 10 institutions. Six centers were located in the United States (US), and an additional four were located in Canada, India, Taiwan, and Turkey. There were five institutions whose responses reflected the practices of >1 clinician.

SRS practices

The number of radiosurgery cases performed at each institution in 2021 ranged from 168 to 741 cases (mean 406) (Table 2). At the US institutions, 25-85.7% of these cases were performed on patients with metastatic tumors, while internationally percentages ranged from 2 to 53%. Gamma knife SRS was used in all 10 institutions, while two of these also offered the option of linear accelerator SRS. Frame-based immobilization was used for 71% of cases. Mask-based immobilization was used for 28% of cases, while <1% used a combination of both frame and mask-based. The majority of brain metastases were irradiated with single-fraction SRS (83%), while 16 % were treated with hypofractionated SRS and <1% used a combination.

**Table 2:** Responses to Survey Questions 2-13 and 17-18 Descriptive statistics on the surveyed center’s SRS volume, type, and machine are included as ranges and means. Additionally, responses regarding SRS and SL-SRS practices are included as percentages of total responses. Abbreviations: US – United States, SRS – stereotactic radiosurgery, GK – Gamma knife, LINAC – linear accelerator, CNS – central nervous system.

Questions	Responses
Total number of cases treated with radiosurgery at your institute last year (2021).	Mean: 406 cases Range: 168-741 cases
Number of brain metastases patients treated with radiosurgery per year.	US Range: 25-85.7% International Range: 2-53%
Does your institution have a formal or informal upper limit on a number of brain metastases treatable with SRS (in 1-5 fractions)? If yes, what is that limit?	60% No limit 40% Upper limit exists, range 15-25 mets
What percentage of your SRS-treated brain metastases patients had this number of lesions treated at the time of SRS?	Average 1-4 lesions: 68.1%, 5-10 lesions: 20.2%, 11-15 lesions: 6.5%, 16-20 lesions: 3.5%, 21+ lesions: 1.6%
Which machine(s) does your institute use for SRS?	GK: 100% of institutions LINAC: 20% of institutions
Percentage of cases treated with frame or mask-based immobilization or combination.	Frame-based cases: 71% Mask-based cases: 28% Combination cases: <1%
Percentage of cases treated with single fraction SRS, hypofractionated SRS, and combined single fraction/hypofractionation in one patient	Single Fraction cases: 83% Hypofractionated cases: 16% Combined cases: <1%
Does your institution use selective lesion SRS?	80% Yes 20% No
Are there any established clinical indications for selected lesion SRS at your institution?	20% Yes 80% No
Are there research protocols in your institution that would take priority over a selected lesion approach for patients with multiple brain metastases?	10% Yes 90% No
Are there clinical trials at your institution that specifically request selected lesion SRS in order to study the effect of a drug in CNS?	10% Yes 90% No
What is your cutoff size (largest dimension, or volume) for SRS for lesions in eloquent areas such as the brainstem or motor cortex?	a) 5mm/0.065cc 0% b) 10mm/0.52cc 0% c) 20mm/4.19cc 20% d) 25mm/8.18cc 10% e) 30mm/14.14cc 60% f) No cutoff 10%
What is your cutoff size for SRS for lesions in non-eloquent areas (largest dimension, or if volumetric cut off, diameter of equivalent circle)?	a) 10mm/0.52cc 0% b) 20mm/4.19cc 0% c) 25mm/8.18cc 0% d) 30mm/14.14cc 30% e) 40mm/33.51cc 60% f) No cutoff 10%

The majority of programs (six out of 10) had no formal or informal upper limit for the number of brain metastases that could be treated by single or fractionated SRS. The range for programs that did report an upper limit was 15-25 brain metastases. Table 2 reports the distribution of the number of lesions treated per SRS session with the majority treating fewer than 10 lesions (88%).

SL-SRS practices

SL-SRS was performed at eight of 10 institutions (five out of six US institutions and three out of four international institutions) (Table 2). However, only two institutions had established clinical indications for SL-SRS (one in the US and one internationally) and one additional program reported clinical trials that require SL-SRS in order to study the efficacy of CNS-penetrating targeted therapies (single US institution). One program reported research protocols for untreated brain metastases that would take priority over SL-SRS (a program outside the US).

**Figure 1:**
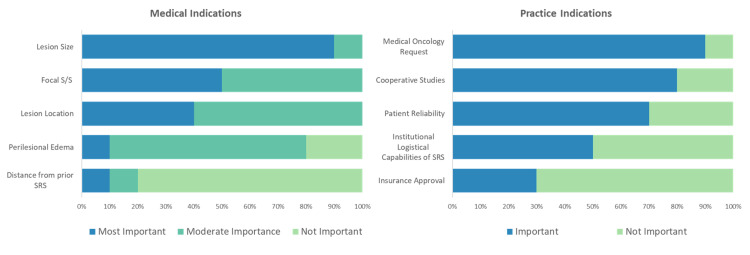
Medical and Practice Indications for SL-SRS Relative importance of medical and practice indications in considering SL-SRS. For medical indications, respondents were asked to rank how they view the importance of each indication, 1-5. Rankings of 1-2 = most important, 3-4 = moderately important, and 5 = least important. For practice indications, respondents designated factors as either important or not. Results are shown as a percentage of total responses. Abbreviations: S/S – signs and symptoms, SRS – stereotactic radiosurgery.

Respondents were asked to rank the importance of five factors that may influence their decision to treat any single lesion in SL-SRS (Figure 1). Ninety percent of respondents ranked size as the most important factor. Next, lesion location and focal signs and symptoms attributable to the lesion were both considered moderately important. Conversely, 80% ranked distance from prior SRS as the least important factor. Perilesional edema was similarly viewed as less important at most programs (90%).

Respondents were asked to comment on size cutoffs for treating lesions in eloquent or non-eloquent areas. Responses are depicted in Table 2 (above). Most institutions (60%) reported that 30 mm (largest dimension) and 14.14 cc (volume) were their cutoffs for treating lesions in eloquent areas, such as the brainstem or motor cortex, while 40 mm and 33.51 cc were the cutoffs for non-eloquent areas. One institution had no size/volume cutoff for eloquent or non-eloquent areas. These cutoffs were not affected by having gamma knife versus linear accelerator-based capabilities.

**Figure 2:**
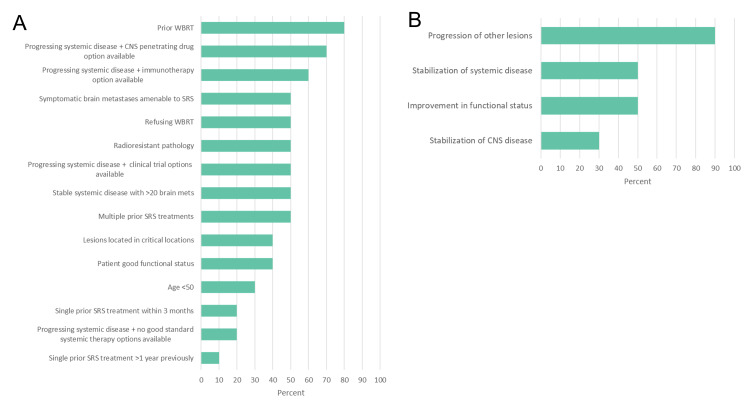
Factors Leading a Clinician to Consider SL-SRS in an Example Case (A) and to Remaining Lesions (B) A) Factors leading a clinician to consider SL-SRS in an example case. Results are shown as a percentage of total responses. B) Factors leading a clinician to consider SL-SRS to remaining lesions not initially treated at a future date. Results are shown as a percentage of total responses. Abbreviations: WBRT – whole-brain radiotherapy, CNS – central nervous system, SRS – stereotactic radiosurgery.

Respondents designated several factors that would encourage them to consider SL-SRS in a hypothetical case of a patient with biopsy-proven carcinoma and >15 previously treated brain metastases (Figure 2A). “Prior WBRT,” “progressing systemic disease and CNS-penetrating drug option available,” and “progressing systemic disease and immunotherapy option available” were the most common responses. The majority of respondents cited “specific request by medical oncology” as well as “cooperative studies in this topic” as factors that might push them toward SL-SRS. Several institutions specified factors beyond the listed options. One institution reported that, for patients with >20 lesions, they treated the largest lesions with SRS and then followed with WBRT, termed the “pre-WBRT boost.” Another institution reported a similar pattern of treating the largest, most symptomatic, or lesions in the most eloquent areas and then followed with WBRT. Poor functional status in patients with an unclear clinical “evolution” was also cited as a factor with plans to follow up in one month, with consideration of additional treatment if the patient recovers systemically.

Finally, when asked which factors would drive clinicians to bring back patients for treatment of the remaining lesions, “progression of other lesions” was the most cited factor (90%, Figure 2B). Fifty percent of respondents also cited “improvement in functional status” and “stabilization of systemic disease” as additional reasons. One institution reported that if patients were symptomatic and the symptoms localized to the untreated lesion, they would consider returning for further treatment. Additionally, one institution reported that they would consider additional treatment at the patient’s request.

## Discussion

Management of brain metastases today requires a multidisciplinary approach. Unlike two decades ago when the treatment options were limited to WBRT, followed by chemotherapy, the introduction of radiosurgery, immunotherapy, and highly CNS-penetrating systemic agents has not only increased the options for successful treatment of brain metastases but has also raised the issue of treatment sequence in order to maximize the duration of efficacy and limit toxicity. While brain metastases clearly remain part of a systemic problem, the brain is a unique site because of functional localization within it making some lesions more critical to treat than others.

To our knowledge, this is the first survey to evaluate SL-SRS practices across multiple institutions worldwide. While the data are qualitative in nature, several insights can be gleaned. Most importantly, SL-SRS is not practiced universally, but the idea is also not new. There is a range of differing practices even within the 10 institutions that responded to the survey. In selecting patients for SL-SRS, prior WBRT was most frequently reported as a factor that would push the provider toward SL-SRS. This trend is concordant with the numerous studies suggesting the efficacy of SRS after WBRT [15-17], as well as the notion that repeat WBRT may not be a good clinical option if lesions progress despite upfront WBRT due to the neurocognitive risks [3,11,18]. SL-SRS to the largest or most symptomatic lesions may be a way to balance disease control with radiotoxicity. Our survey did find that respondents cited size and symptomatology to be the main reasons to treat any lesions when contemplating SL-SRS.

Our survey results also highlight the possibility of using SL-SRS first, followed by WBRT. Often systemic treatments need to be prioritized in patients with rapidly progressing systemic disease. For select respondents, SL-SRS was used upfront for more rapid targeting of higher-risk lesions to enable faster initiation of systemic therapy. In this way, SL-SRS can be used as a pre-WBRT option for patients who will likely need more comprehensive CNS radiotherapy but may have a significant systemic disease burden. Whereas concurrent chemotherapy and WBRT are typically not performed due to risks of toxicity, there is greater flexibility with concurrent SL-SRS and chemotherapy. When the patient is stable from a systemic standpoint, WBRT can be considered for the completion of comprehensive CNS treatment. Additionally, this approach offers the option of evaluating whether WBRT can be deferred if the untreated brain metastases after SL-SRS remain stable or respond to systemic therapy.

The other factors that respondents chose in considering SL-SRS were the progression of systemic disease and the availability of CNS-penetrating drugs or immunotherapies. Again, there are several recent studies suggesting improved outcomes of disease control when combining immunotherapy and SRS across several metastatic disease types compared with the use of either alone [19-22]. In this way, small asymptomatic lesions can be left un-irradiated if CNS-penetrating drugs are available. An example of a case where SL-SRS might be considered could include a patient with lung adenocarcinoma with a standard L858R epidermal growth factor receptor (EGFR) mutation with multiple brain metastases of varying sizes and locations. This patient could be treated with SL-SRS for concerning lesions and then started on the third-generation tyrosine kinase inhibitor (TKI) osimertinib for the management of the remaining brain metastases. Alternatively, a patient with very slowly progressive Her2-positive breast cancer declining WBRT and starting fam-trastuzumab-deruxtecan-nxki (ENHERTU) could be considered for SL-SRS deferring salvage radiation for a time when the additional lesions might progress. Importantly, however, these agents are currently undergoing investigation and are not equally or uniformly effective; thus, surveillance imaging should be closely monitored for progression [23-24].

There are several potential advantages to using SL-SRS, rather than WBRT, as first-line treatment of multiple brain metastases. It allows avoidance of the CNS toxicity of WBRT. More importantly, though, it does not preclude subsequent WBRT or additional SRS. SL-SRS also can be delivered much more quickly than WBRT and can reduce the delay in starting systemic therapy. Because of this last advantage, SL-SRS might be paired safely with newer systemic therapies whose CNS-penetrating capability is untested or unclear and could be extended to use in clinical trials allowing more patients, who would otherwise be excluded, to be enrolled regardless of the presence of brain metastases [25-26].

Finally, without consensus on indications, potential downsides of SL-SRS, however, should also be noted. Importantly, not immediately treating some brain metastases may place the patient at risk for metastatic growth and development of associated symptomatology if the choice of lesions selected is not done with cerebral functional locations in mind. Additionally, metastases from primaries, such as melanoma or renal cells left un-irradiated, may lead to hemorrhage, resulting in functional deficits [27]. Finally, from a practical standpoint, the possibility that subsequent salvage radiosurgical procedures needed thereafter will not be authorized by a third-party payer may also be a real concern.

While this study provides important insight into the current perspectives on SL-SRS, these findings must be viewed in the context of the study’s limitations - which include its survey design, small sample size, low response rate, and reliance on qualitative data. Nonetheless, it offers a unique insight into the developing practice patterns on SL-SRS and shows that there seems to be some commonality despite disparate settings and patient populations. Although there is no clear practice consensus at this time, the fact that SL-SRS exists beyond a single institution may indicate that there is generalizability of these findings. A large retrospective cohort study concentrated on both survival and local CNS control outcomes comparing best standard practice upfront (WBRT or all lesion SRS) with SL-SRS may help clarify and consolidate the indications for this strategy.

More intriguingly, with several institutions now reportedly using SL-SRS, this practice may additionally provide the basis for a unique opportunity. As new targeted immunotherapies are being designed and approved at an increasingly rapid pace, there is an increased need for understanding the CNS efficacy of each of these drugs [25-26,28]. Incorporating SL-SRS into clinical trials of these agents may not only allow for patients with brain metastases to enter more traditional clinical trials of new agents but would also facilitate the safe study of the effect of these agents on small and asymptomatic brain metastases, which could then inform clinical practice and the subsequent formation of guidelines for SL-SRS.

## Conclusions

In our opinion, the use of CNS-penetrating treatments is revolutionizing how we view the treatment of CNS metastasis. Focal therapy carries unique complications that could be deferred if only select lesions are treated. This survey shows that patients with >10-15 brain metastases who have had prior WBRT, those with progression of extracranial disease still with acceptable systemic treatment options available, and those with poor functional status are currently being considered for SL-SRS in institutions worldwide. Larger lesion size, functionally eloquent location, and presence of symptomatology are the most common reasons to choose any given lesion for SRS. Future studies evaluating outcomes after SL-SRS are needed to form a consensus on how to best implement SL-SRS into standard practice. In addition, consideration of this technique for incorporation into new drug clinical trials may allow brain metastases more access to new agents and provide data for each drug’s CNS efficacy.
